# Liposomes Loaded with the Proteasome Inhibitor Z-Leucinyl-Leucinyl-Norleucinal Are Effective in Inducing Apoptosis in Colorectal Cancer Cell Lines

**DOI:** 10.3390/membranes10050091

**Published:** 2020-05-03

**Authors:** Katia Cortese, Silvia Marconi, Cinzia Aiello, Maria Cristina Gagliani, Serena Pilato, Romina Zappacosta, Antonella Fontana, Patrizio Castagnola

**Affiliations:** 1 DIMES, Department of Experimental Medicine, Human Anatomy, University of Genoa, 16132 Genoa, Italy; cortesek@unige.it (K.C.); silviamarconi.sm@libero.it (S.M.); gagliani@unige.it (M.C.G.); 2 IRCCS Ospedale Policlinico San Martino, UO Bioterapie, 16132 Genoa, Italy; cinzia.aiello@hsanmartino.it; 3 Department of Pharmacy, “G. d’Annunzio” University of Chieti-Pescara, 66100 Chieti, Italy; serena.pilato@unich.it (S.P.); r.zappacosta@unich.it (R.Z.)

**Keywords:** liposomes, colorectal cancer, ERBB1, cetuximab, proteasome

## Abstract

Colorectal cancer (CRC) is one of the main causes of cancer-related death in developed countries. Targeted therapies and conventional chemotherapeutics have been developed to help treat this type of aggressive cancer. Among these, the monoclonal antibodies cetuximab (Cxm) and panitumumab specifically target and inactivate the signaling of ERBB1 (EGF receptor), a key player in the development and progression of this cancer. Unfortunately, these antibodies are effective only on a small fraction of patients due to primary or secondary/acquired resistance. However, as ERBB1 cell surface expression is often maintained in resistant tumors, ERBB1 can be exploited as a target to deliver other drugs. Liposomes and immunoliposomes are under intensive investigation as pharmaceutical nanocarriers and can be functionalized with specific antibodies. In this study, we first investigated the anti-cancer activity of a cell permeable tripeptide, leucine-leucin-norleucinal (LLNle), an inhibitor of gamma-secretase and proteasome, in three different CRC cell lines that express ERBB1. We formulated LLNle-liposomes and Cxm-conjugated LLNle-loaded liposomes (LLNle-immunoliposomes) and evaluated their efficacy in inhibiting cell survival. Despite similar pro-apoptotic effects of free LLNle and LLNle-liposomes, immunoliposomes-LLNle were significantly less effective than their unconjugated counterparts. Indeed, immunoliposomes-LLNle were readily internalized and trafficked to lysosomes, where LLNle was likely trapped and/or inactivated. In conclusion, we demonstrated that LLNle was readily delivered to CRC cell lines by liposomes, but immunoliposomes-LLNle failed to show significant anti-cancer activity.

## 1. Introduction

Liposomes are colloids formed by the enclosure of aqueous core into a spherical shaped phospholipidic bilayer. Liposomes, compared to other nanocarriers, have different advantages: (i) are able to solubilize both hydrophilic and lipophilic molecules in the aqueous core and the phospholipidic bilayer, respectively, (ii) are biocompatible because they are derived from naturally occurring phospholipids and cholesterol, (iii) their stability and capacity to deliver drugs can be tuned by choosing the proper composition and preparation procedure [1], (iv) they can be engineered in order to favor their targeting [2] or (v) they are supported in order to provide a system that is more suitable for clinical applications [3]. For all these reasons they have been extensively used as pharmaceutical nanocarriers in various clinical applications [1,4].

Leucine-leucin-norleucinal (LLNle) is a cell permeable peptide inhibitor of gamma-secretase and proteasome, which triggers cell death in glioblastoma tumor-initiating cells [5,6] and precursor-B acute lymphoblastic leukemia [6]. In particular, LLNle is effective versus cancer cells because these display proteotoxic stress and therefore are more sensitive to proteasome inhibition, which causes a great accumulation of misfolded proteins. However, gamma-secretase and proteasome inhibitors are not devoid of adverse effects on normal cells. It is well known that the former inhibitors cause acute gastrointestinal toxicity [7] while for the latter, cardiovascular toxicity and peripheral neuropathy are frequently reported [8].

Colorectal cancer (CRC) is one of the main causes of cancer related death in developed countries [9,10]. Among the drugs employed for CRC therapy are the monoclonal antibodies cetuximab (Cxm) and panitumumab, which specifically target and inactivate the signaling of ERBB1, which is the EGF receptor. Unfortunately, these antibodies are effective only on a small percentage of patients due to primary or secondary/acquired resistance to this therapy [11]. However, ERBB1 cell surface expression may be maintained when the cells become resistant and therefore this can be exploited as a target to deliver other drugs [12,13].

It has already been demonstrated [14] that the combination of cytotoxic agents with immunotargeting, either directly conjugated or in drug carriers such as liposomes, reduces side effects and results in enhanced and more localized impact than the drug alone. To name a few examples, immunoliposomes functionalized with Cxm have already been investigated. In particular, it was reported that these Cxm-liposomes were able to inhibit skin cancer cell growth and to improve patient compliance by delivering the highly hydrophilic chemotherapeutic 5-fluorouracil (5-FU). This delivery system was shown to favor skin penetration [15]. Indeed, immunoliposomes carrying a combination of Cxm and Oxaliplatin (L-OH), a platinum derivative with good tolerability, demonstrated efficacy and increased sensitivity in colorectal cancer (CRC) cell lines [16]. More specifically, the efficacy of this system was more than three times compared to the free or non-immunoliposomal delivery system in wild-type KRAS metastatic CRC expressing ERBB1.

In the present study, we intended to evaluate a novel combination of Cxm with the proteasome inhibitor tripeptide LLNLe in order to limit its toxicity towards normal cells improving cancer cell targeting efficiency using three different CRC cell lines expressing the ERBB1 receptor.

## 2. Materials and Methods 

### 2.1. Materials 

1-Palmitoyl-2-oleoyl-sn-glycero-3-phospho-coline (POPC), cholesterol (Chol, >98% purity), 1,2-distearoyl-sn-glycero-3-phospoethanolamine-*N*-[methoxy-(polyethylene glycol)-2000] (ammonium salt) (DSPE-PEG_2000_), 1,2-distearoyl-sn-glycero-3-phosphoethanolamine-*N*-[maleimide(polyethylene glycol)-2000] (ammonium salt) (DSPE-PEG_2000_-Mal), were purchased from Avanti Polar Lipids (700 Industrial Park Dr, Alabaster, AL 35007, USA). Chloroform, leucine-leucin-norleucinal, z-LLNle-CHO (LLNle), 2-iminothiolane hydrochloride (Traut’s Reagent), Bradford protein assay kit, Whatman Nucleopore track-etch membranes, Sephadex G-50 and G-25 were obtained from Merck KGaA (Darmstadt, Germany). 

### 2.2. Instruments 

The extrusion was performed on a nitrogen-driven Lipex Biomembranes (Vancouver, BC, Canada) apparatus operating at room temperature. UV/Vis absorption measurements were performed on Jasco V-550 UV/Vis (Cremella, Italy). Measurements of DLS and Zeta potential were performed by using a 90 Plus/BI-MAS ZetaPlus multiangle particle size analyzer (Brookhaven Instruments Corp., Holtsville, NT, USA) on diluted samples.

### 2.3. Liposomes Preparation 

Two pegylated liposomal formulations were used in the present study to prepare LLNle-enriched liposomes and immunoliposomes. A chloroform solution of lipids consisting of POPC, Chol, DSPE-PEG_2000_ (85.9:9.1:5) [17], in the absence or in the presence of DSPE-PEG_2000_-Mal at 1.2 mol % of total lipid [18], was dried to a thin film in a round bottom flask on a rotary evaporator at 40 °C and then further dried for 2 h under vacuum. After the addition of the phosphate-buffered saline (PBS, pH 7.4), the resulting multilamellar vesicle suspension was extruded 5 times through a polycarbonate filter with a pore size of 100 nm using a Lipex Biomembranes extruder. The conjugation of tripeptides to the liposomal surface was achieved by adding a solution of 6 mM LLNle in DMSO to the suspension at a POPC to protein molar ratio of 70:30 for 10 min at room temperature [19]. This concentration of LLNle solution allowed us to limit the concentration of DMSO at 2.5% *v*/*v*. For the preparation of immunoliposomes, Cxm was first thiolated for 1 h at room temperature by reacting with a 10-fold excess of Traut’s reagent (2-iminothiolane) 2 mg/mL in PBS buffer. Unreacted Traut’s reagent was removed using a Sephadex G-50 column pre-equilibrated with PBS and the resulting solution of thiolated antibody was stored at 4 °C. The coupling reaction was run adding thiolated Cxm to the liposomes containing maleimide-terminated linker at an IgG to phospholipid molar ratio of 1:2000, under a controlled atmosphere for 18 h at room temperature [12]. Uncoupled Cxm was separated from the immunoliposomes by passing the coupling mixture through a Sephadex G-25 column in PBS buffer. The effective amount of Cxm conjugated with liposomes was calculated, with an indirect method, as the difference between the initial amount of antibody used for the incubation of liposomes and the quantity of free Ab retained in the column. The uncoupled Cxm was recollected with a washing step of the column and a Bradford protein assay was performed in order to determine the coupling efficiency. The Cxm content was measured spectrophotometrically at λ 595 nm on a solution of 50 µL of the eluted solution in the presence of 1.5 mL of Bradford Reagent. All the samples were freeze-dried in aliquots to ensure stability until use. Size and ζ-potential of liposomes and immunoliposomes were determined by dynamic light scattering measurements after each step of extrusion, incubation with LLNle, conjugation n of Cxm and final gel filtration with Sephadex G-25.

### 2.4. Antibodies and Chemicals

The anti-polyUbiquitinated antibody (clone FK1) was acquired from Merck Millipore (Burlington, MA, USA); the anti-PARP antibody (#9542) was purchased from Cell Signaling (Danvers, MA, USA); the anti-ERBB1 antibody (sc-03) was from Santa Cruz Biotechnology Inc. (Santa Cruz, CA, USA); the anti-tubulin (clone B-5-1-2) was acquired from Sigma-Aldrich. The Cxm antibody was obtained from Merck KGaA (Darmstadt, Germany). The monoclonal antibodies anti-LAMP1 (H4A3) were obtained from the Developmental Studies Hybridoma Bank. LysoTracker™ Red DND-99, Alexa-549 anti-mouse IgG antibodies, Alexa-647 conjugated anti-human IgG antibodies, DAPI and Alexa-488 conjugated acetylated low-density lipoprotein (L-23380) were all acquired from Thermofisher Scientific (Waltham, MA, USA). DMSO was purchased from Sigma-Aldrich (St.Louis, MO, USA).

### 2.5. Cell Cultures

CRC cell lines Caco-2 and LoVo were obtained from Banca Biologica and Cell Factory in IRCCS Ospedale Policlinico San Martino, belonging to the European Culture Collection’s Organization. GP2d cells were obtained from the European Collection of Authenticated Cell Cultures (ECACC) (Porton Down Salisbury, UK). Cells were cultured according to the manufacturer’s instructions. Where indicated, cells were challenged with LLNle while control cultures were challenged with DMSO (used as a solvent for LLNle).

### 2.6. Cell Survival Assay

Cells were plated in 24-well plates in complete medium and challenged with liposomes, LLNle or DMSO. Treatments were administered for 72 h. Cell survival was measured using the 3-(4,5-dimethylthiazol-2yl)-2,5-diphenyltetrazolium bromide (MTT) (Sigma-Aldrich, 14508 Saint Louis, MO, USA) colorimetric assay and the AD 200 plate reader from Beckman Coulter Inc. (Brea, CA, USA).

### 2.7. Immunofluorescence Analysis 

Cells were treated for up to 24 h with indicated reagent, fixed in 3% paraformaldehyde (PFA), 2% sucrose in phosphate-buffered saline (PBS) pH 7.4. Cell permeabilization was performed with triton and goat serum (Sigma-Aldrich) was used as a blocking reagent. Anti-human or anti-mouse Alexa-conjugated secondary antibodies were used to reveal primary antibodies. Image deconvolution and acquisition was performed with an Axio Imager A2M microscope equipped with an apotome module (Carl Zeiss, Jena, Germany).

### 2.8. Flow Cytometry 

Metabolic active, apoptotic and necrotic cells were evaluated by using the Vybrant^®^ Apoptosis Assay Kit (Thermo Fisher Scientific, Waltham, MA, USA) with a minor procedure modification as we used the nuclear staining fluorochrome sytox blue instead of the sytox green. Cells were then analyzed using a cyan ADP flow cytometer (Beckman Coulter, Brea, CA, USA). 

### 2.9. Immunoblot Analysis

Cells were lysed using lysis buffer (hepes pH 7.4 20 mM, NaCl 150 mM, 10% glycerol, 1% triton X-100) with protease inhibitors cocktail complete (Roche Applied Science, Penzberg, Germany) and sodium orthovanadate or Phostop (Roche). Proteins were resolved using SDS-polyacrylamide gel electrophoresis and blotted on PVDF (Merck Millipore) membranes. Detection was performed using ECL detection reagent (BioRad, Hercules, CA, USA) according to the manufacturer’s protocol. ECL signals were detected, recorded using the Uvitec Cambridge gel doc system and software (Cambridge, UK).

### 2.10. TEM Analysis

Electron microscopic analysis on liposomes preparations was performed as follows. After resuspension in 20 μL PBS (pH 7.4), fixation was performed by adding an equal volume of 2% paraformaldehyde in 0.1 mol/L phosphate buffer (pH 7.4). Liposomes were then adsorbed for 20 min to formvar-carbon coated copper grids by floating the grids on 5 μL drops on parafilm. Subsequently, grids were rinsed in PBS and negatively stained with 2% uranyl acetate for 5 min at room temperature. Stained grids were embedded in 2.5% methylcellulose for improved preservation and air dried before examination. Electron micrographs were taken using a TEM microscope (HT7800 series, Hitachi, Tokyo, Japan) equipped with a Megaview 3 digital camera and Radius software (EMSIS, Münster, Germany).

### 2.11. Statistical Analysis

This analysis was performed using Prism (GraphPad Software, La Jolla, CA, USA). In particular, we used the one-way ANOVA plus post-hoc Tukey’s multiple comparison test. Mean differences were considered statistically significant (*p*-value) at *p* < 0.05. Details of the results of the statistical analysis are provided in Table S1.

## 3. Results and Discussion

### 3.1. LLNle Inhibits Cell Survival and Induces Apoptosis in CRC Cell Lines 

**Figure 1:**
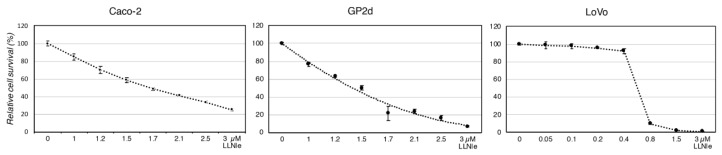
LLNle causes a concentration-dependent inhibition of survival in Caco-2, GP2d and LoVo cell lines. An MTT assay was performed after 72 h of treatment of the indicated cell culture, *n* = 4 replicas were performed and average values and SD are shown for each concentration indicated on the *x*-axis. An interpolation curve of survival values is shown for the GP2d cell line.

To test the hypothesis that LLNle was able to inhibit CRC cell survival by inducing apoptosis, similarly to what we observed in Glioblastoma (GBM) cells, we performed an MTT assay after 72 h of treatment with several concentrations of this substance. The Caco-2 and GP2d cell lines showed a similar smooth concentration-dependent response to LLNle with concentrations able to obtain a 50% inhibition of cell survival in the low micromolar range of 1.5–1.7 µM (Figure 1). 

**Figure 2:**
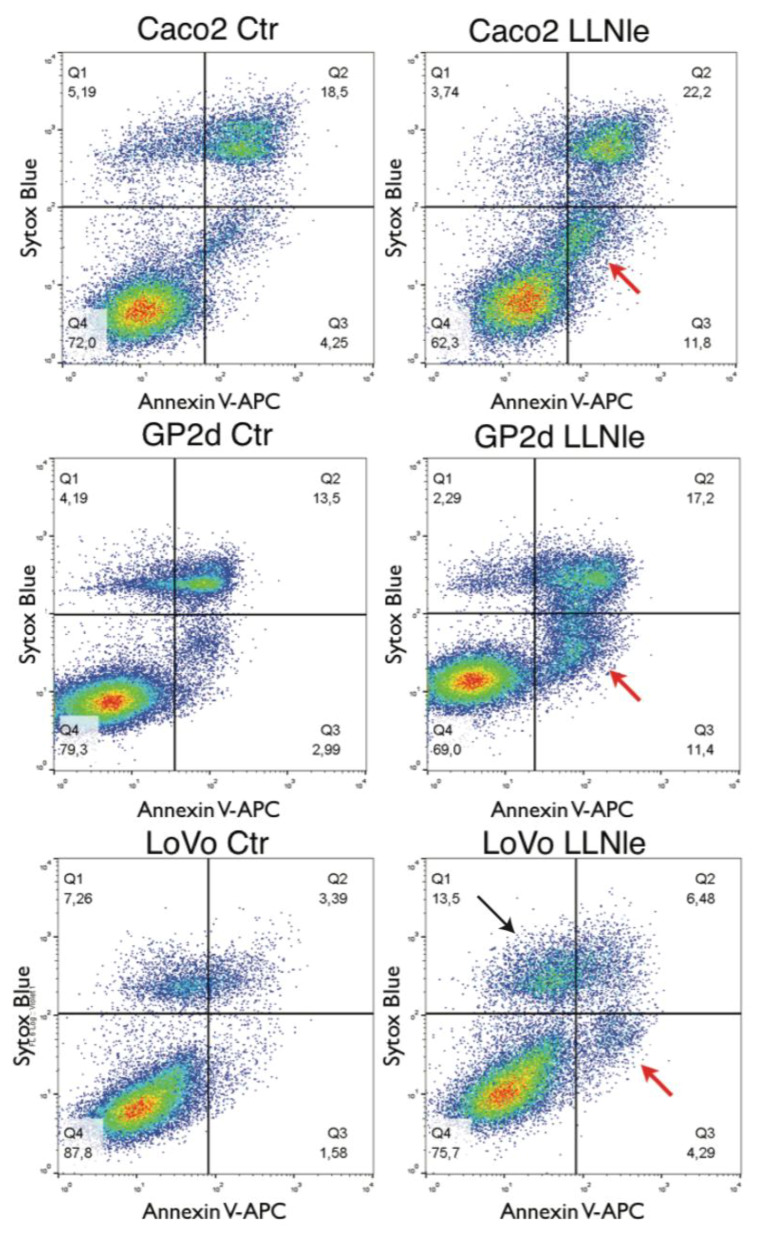
LLNLe induces apoptosis in Caco-2, GP2d and LoVo cell lines. Cells were treated for 72 h with LLNle (1.7, 1.4 and 0.6 µM LLNle for Caco-2, GP2d and LoVo, respectively) or with DMSO used as a control. Cells were labeled with both the DNA-specific and cell-impermeant sytox-blue, and annexin-V APC and fluorescence emissions were analyzed by FCM. Early apoptotic (annexin-V APC^+^ and sytox-blue^−^) cells are indicated by red arrows while necrotic cells (annexin-V APC^−^ and sytox-blue^+^) are indicated by a black arrow.

Instead, the LoVo cell line showed a sharp increase in survival inhibition ranging from 0.4 to 0.8 µM LLNle with a calculated concentration of 0.6 µM able to yield a 50% inhibition of cell survival. To verify whether apoptosis was induced by this substance in these CRC cell lines, we performed an apoptosis assay able to identify by flow cytometry early and late apoptotic cells along with necrotic cells. We indeed observed in the three CRC cell lines treated with LLNle (1.7, 1.4 and 0.6 µM LLNle for Caco-2, GP2d and LoVo, respectively) an increase of early apoptotic cells compared to DMSO controls. In addition, in LoVo cells, we also detected an increase of necrotic cells (Figure 2). Taken together, LLNLe shows pro-apoptotic activity in CRC cell lines, either free o encapsulated within liposomes.

### 3.2. Liposomes Generation and Chemical-Physical-Ultrastructural Characterization

As shown in Table 1; Table 2, after the extrusion, all liposomal preparations showed similar sizes of ~170 nm, and ζ-potential of ~−22 mV.

**Figure 3:**
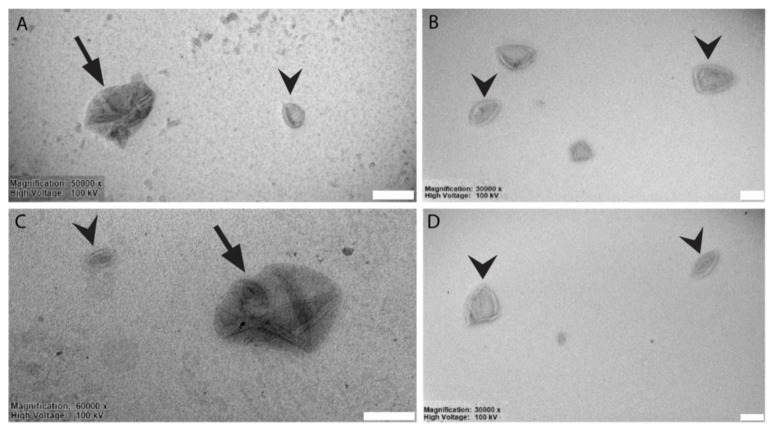
Representative TEM micrographs of liposomes visualized by negative staining. (**A**) immunoliposomes-LLNle (arrows indicate aggregates; arrowheads single liposomes), (**B**) immunoliposomes, (**C**) LLNle-liposomes, (**D**) liposomes. Arrows: aggregates of liposomes; arrowheads single liposomes. Bars = 200 nm.

A minimal increase in average diameter after Cxm conjugation was observed, confirming the functionalization of uncoupled maleimide groups with the Ab. During the last step of gel filtration, with the likely retention of not covalently bound Ab in the column, there was a slight reduction of liposomal mean diameter. As lyophilization is generally used in order to allow the facile conservation of the liposomal dispersion, the dimensions of liposomes after the reconstitution of freeze-dried power with the proper amount of MilliQ water were also monitored. Indeed, the size of liposomes slightly increased as a consequence of the freeze-drying and rehydration processes. Despite the presence of larger aggregates, a second population of liposomes, with dimensions of 200–300 nm and still negative surface charge, was preserved in the samples. To better characterize the different liposomes conjugates, we performed negative staining for TEM imaging of a suspension of these objects. The ultrastructural analysis showed that liposomes had an average diameter of ~170–200 nm and displayed the characteristic cup-shaped morphology (Figure 3, arrowheads). This feature usually represents a fixation artifact, due both to the interaction between the sample and the negative stain and the alteration induced by the drying step. We also report that, occasionally, larger aggregates (>200 nm) were observed in both LLNle liposomes and LLNLe immunolipososomes populations (Figure 3A–C, arrows). Collectively, the ultrastructural analysis is in good agreement with DLS and Zeta potential analysis.

### 3.3. LLNle-Liposomes Reduce Cell Survival of CRC Cell Lines and Show Higher Inhibition Activity Compared to Immunoliposomes-LLNle

**Figure 4:**
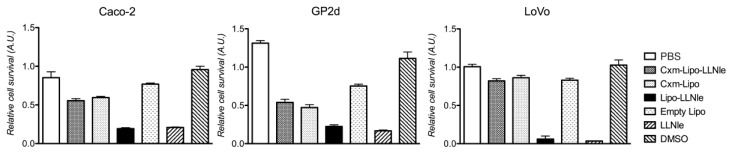
LLNle-liposome inhibits CRC cell line survival. MTT analysis of Caco-2, GP2d, and LoVo cell line treated with LLNle, DMSO (used as a control for LLNle), LLNle-liposomes, Cxm-liposomes-LLNle, empty liposomes, and PBS (control for liposomes) for 72 h. LLNle-liposomes were diluted to achieve an LLNle final concentration in the culture medium equivalent to 2.5, 2.0 and 0.8 µM of free LLNle for Caco-2, GP2d and LoVo, respectively. All measurements here reported are presented as mean ± standard deviations (s.d.), n = 4. Details of the results of the statistical analysis are provided in Table S1.

To establish whether liposomes loaded with LLNle were able to inhibit in vitro CRC cell survival and induce apoptosis, we challenged Caco-2, GP2d and LoVo cell lines with them. In addition, we wanted to assess whether these liposomes would have been able to specifically target CRC cells expressing the ERBB1 receptor when conjugated with Cxm, which was the humanized anti ERBB1 receptor monoclonal antibody used in CRC therapy. Our experiments showed that both LLNle-liposomes and an equivalent concentration of free LLNle were able to achieve a survival inhibition of about 80% (2.5, 2.0 and 0.7 µM LLNle for Caco-2, GP2d and LoVo, respectively) and were able to significantly inhibit cell survival (*p* < 0.001) in the three cell lines tested, compared to medium supplemented with PBS or DMSO treated controls, respectively. However, no statistically significant difference in cell survival was observed between LLNle-liposomes and free LLNle (Figure 4). Moreover, we observed that Cxm-conjugated liposomes and Cxm-liposomes-LLNle, used at the same final concentration of LLNle-liposomes, caused a statistically significant reduction of cell survival compared to PBS control (*p* < 0.001) but the extent of this reduction was significantly less than that obtained with LLNle-liposomes. Strikingly, no statistically significant difference in survival was observed when the two immunoliposomes were compared (Figure 4). Taken together, these results demonstrate that LLNle, when conveyed via Cxm-conjugated liposomes is ineffective on cell survival. Details of the statistical analysis are provided in Table S1.

### 3.4. LLNle-Liposomes Inhibit Proteasomal Degradation and Induce Apoptosis in CRC Cell Lines in a Fashion Similar to Free LLNle

**Figure 5:**
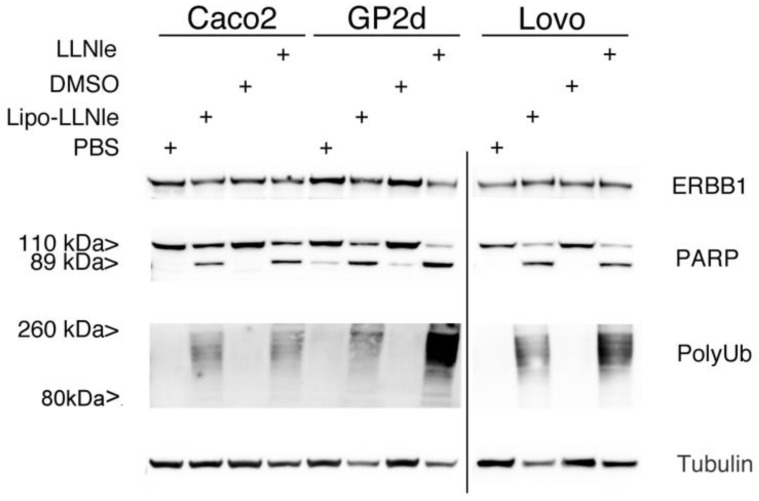
LLNle-liposome inhibits proteasomal degradation of poly-ubiquitinated protein and induces apoptosis in CRC cell lines. Immunoblot analysis of whole-cell lysates of Caco-2, GP2d, and LoVo cell lines treated with LLNle, DMSO (used as control for LLNle), LLNle-liposomes, and PBS (control for LLNle-liposomes) for 72 h. LLNle-liposomes were diluted to achieve an LLNle final concentration in the culture medium equivalent to 1.7, 1.4 and 0.6 µM LLNle of free LLNle for Caco-2, GP2d and LoVo, respectively. All three cell lines display expression of ERBB1, the EGF receptor. A high molecular weight smear signal, corresponding to poly-ubiquitinated proteins, and the generation of the 89 kDa isoform of the protein PARP was detected by specific Ab only in LLNle-liposomes and LLNle treated samples. Tubulin was used as a loading control.

To verify whether the mechanism of action of LLNle-liposomes at the basis of the inhibition of cell survival was identical to that demonstrated for the free LLNle (e.g., inhibition of the proteasome and apoptosis), we investigated the occurrence of these processes in the three cell lines used in this study. In these experiments, we used 1.7, 1.4 and 0.6 µM LLNle for Caco-2, GP2d and LoVo, respectively and LLNle-liposomes were diluted in order to obtain the same final concentration of LLNle in the culture media. In particular, we analyzed by immunoblot the accumulation in cell lysates of poly-Ubiquitinated proteins and the cleavage of the poly adenosine ribonucleotide polymerase (PARP) as a read out of proteasome inhibition and apoptosis, respectively. This analysis showed that LLNle-liposomes induced a pattern of high molecular weight protein polyubiquitination and the appearance of the 89 kDa PARP specific band was very similar to the one displayed by free LLNle (Figure 5). These results confirmed that LLNle was responsible for the survival inhibition and apoptosis observed in these cells.

### 3.5. Immunoliposomes-LLNle Are Directed to the Endosomal/Lysosomal Compartment

**Figure 6:**
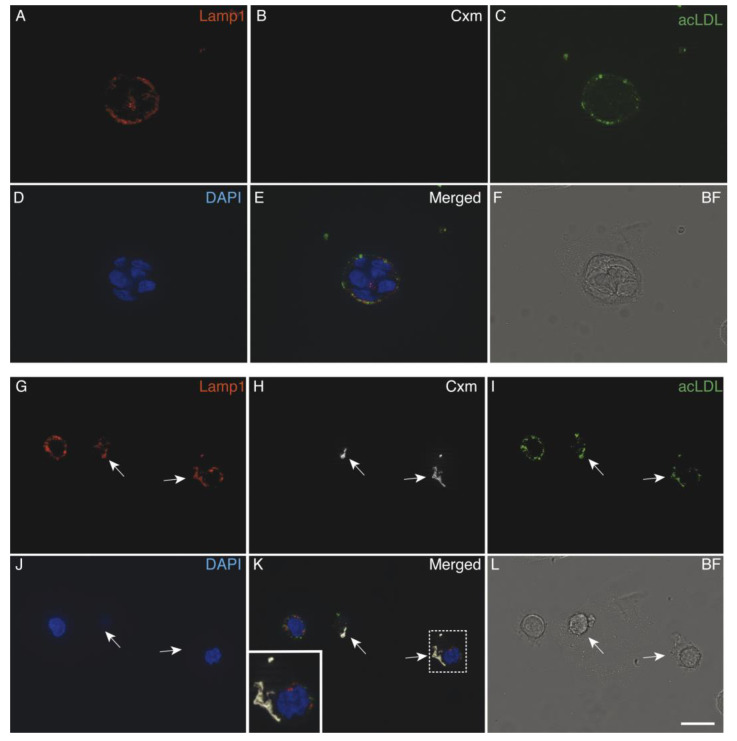
Cxm-Liposomes are endocytosed and trafficked to the endo/lysosomal compartment. Immunofluorescence analysis of the Caco-2 cell line treated for 2 h with a PBS control (**A**–**F**) or with Cxm-LLNle (**G**–**L**) and acLDL. Anti LAMP1 Ab and DAPI were used to detect lysosomes and nuclei, respectively. Arrows point to regions of colocalization of LAMP1, Cxm and acLDL signals. In the boxed area is shown a digitally enlarged area of triple colocalization. A single channel along with merged and bright-field images are shown. Bar = 20 µm.

We previously showed that immunoliposomes-LLNle had poor anti-survival activity (Figure 4). To investigate the possible reasons for this result, we first ruled out that the minor anti-survival activity of the immunoliposomes-LLNle was due to the loss of ERBB1 expression by our CRC cell line models. To this aim, we performed an immunoblot analysis which showed that this was not the case as ERBB1 was detected in all the cell lines (Figure 6). This result suggests that alternative mechanisms are interfering with immunoliposomes-LLNle activity. We, therefore, hypothesized that the binding of Cmx immunoliposomes (loaded or unloaded with LLNle) to the ERBB1 receptor might result in endocytosis and trafficking to the endo/lysosome compartment. To test this, we performed an immunofluorescence analysis using acetylated low-density lipoprotein (acLDL) to reveal the endocytic compartment, lysotracker or the lysosomal-associated membrane protein (LAMP1) as lysosome markers, and fluorescent anti-human antibodies to detect Cxm-conjugated liposomes. Indeed, the immunofluorescence analysis showed that immunoliposomes are readily internalized within cells and localized to lysosomal compartments. Figure 6 shows representative images that show colocalizations of Cxm with both acLDL and lysotracker or LAMP1 in the Caco-2 cell lines. In control cultures, not treated with liposomes, the staining with anti-human antibodies was not observed. These results show that LLNle might be either trapped and unable to reach the proteasomes or, less likely, somehow inactivated/degraded. Consistently, we obtained similar results with both GP2d cells and LoVO cells after 2 h of treatment with immunoliposomes-LLNle diluted in order to obtain 1.4 µM LLNle in the culture media (Supplementary Figures S1 and S2).

Taken together, our results strongly suggest that LLNle, when delivered with immunoliposomes, is either trapped or inactivated within lysosomes due to endocytic trafficking. This was actually unexpected, because we hypothesized that LLNle, being liposoluble, could diffuse to proteasomes. Instead, lysosomal localization is probably interfering with the interaction of LLNle with proteasomes. Notwithstanding, as a positive note, immunoliposomes may be used as effective carriers of pro-drugs or drugs which have as specific targets the lysosomes, or that are insensitive to the lysosomal microenvironment. Further studies are required to demonstrate that lysosome-specific drugs may be useful for cancer therapy when delivered with immunoliposomes and show less toxic profiles towards normal cells.

## 4. Conclusions

In the present study, we evaluated a novel combination of Cxm with the proteasome inhibitor tripeptide LLNLe in order to limit its toxicity towards normal cells improving cancer cell targeting efficiency using three different CRC cell lines expressing the ERBB1 receptor. We demonstrated that LLNle is readily delivered to CRC cell lines by liposomes and exerts pro-apoptotic activity. We also demonstrated that immunoliposomes can be internalized by these cells and trafficked to lysosomes, which unfortunately limited their efficacy in terms of cell survival inhibition. Therefore, our main goal of cell survival inhibition using immunoliposomes-LLNle was not achieved but immunoliposomes might be useful to deliver drugs to lysosomes as a specific target.

## Figures and Tables

**Table 1:** Dimensions (mean diameter, nm) of the investigated liposomes monitored at different steps of protocol preparation.

Protocol Step	Liposomes	LLNle-Liposomes	Cmx-Liposomes	Cmx-Liposomes-LLNle
Extrusion	170.7 ± 26.5	170.7 ± 26.5	176.1 ± 12.1	176.1 ± 12.1
LLNle incubation	-	189.4 ± 13.5	-	189.5 ± 3.0
Cxm conjugation	-	-	187.1 ± 7.3	199.9 ± 8.7
G-25 filtration	143.7 ± 10.0	183.1 ± 16.0	165.6 ± 7.2	162.7 ± 8.8
Rehydration after freeze-drying ^1^	587.5 ± 7.6 221.6 ± 26.1	479.6 ± 6.7 171.3 ± 27.5	815.4 ± 267.0 285.6 ± 28.8	1198 ± 63 291.9 ± 13.6

^1^ Dimensions of the two populations monitored.

**Table 2:** ζ-potential (mV) of investigated liposomes monitored at different steps of protocol preparation.

Protocol Step	Liposomes	LLNle−Liposomes	Cmx−Liposomes	Cmx−Liposomes−LLNle
Extrusion	−23.3 ± 0.6	−23.3 ± 0.6	−22.9 ± 2.8	−22.9 ± 2.8
LLNle incubation	-	-	-	-
Cxm conjugation	-	-	−20.0 ± 1.1	−22.2 ± 4.3
G−25 filtration	−23.6 ± 1.6	−21.4 ± 0.5	−20.3 ± 0.2	−20.9 ± 3.5
Rehydration after freeze−drying	−26.1 ± 2.9	−27.9 ± 2.1	−23.1 ± 2.2	−29.2 ± 8.8

## Supplementary Materials

The following are available online at https://www.mdpi.com/2077-0375/10/5/91/s1, Table S1: results of statistical analysis, Figure S1: Immunofluorescence analysis of the GP2d cell line treated for 2 h with PBS control (A–F) or with Cxm-LLNle (G–L) and Figure S2: Immunofluorescence analysis of the LoVo cell lines treated for 2 h with PBS control (A–F) or with Cxm-LLNle (G–L).
