# Supplementary figures and images for: Liposomes Loaded with the Proteasome Inhibitor Z-Leucinyl-Leucinyl-Norleucinal Are Effective in Inducing Apoptosis in Colorectal Cancer Cell Lines
Source: Membranes (Basel). 2020 May 3;10(5):91. doi: 10.3390/membranes10050091 (PMC7281214; doi:10.3390/membranes10050091)

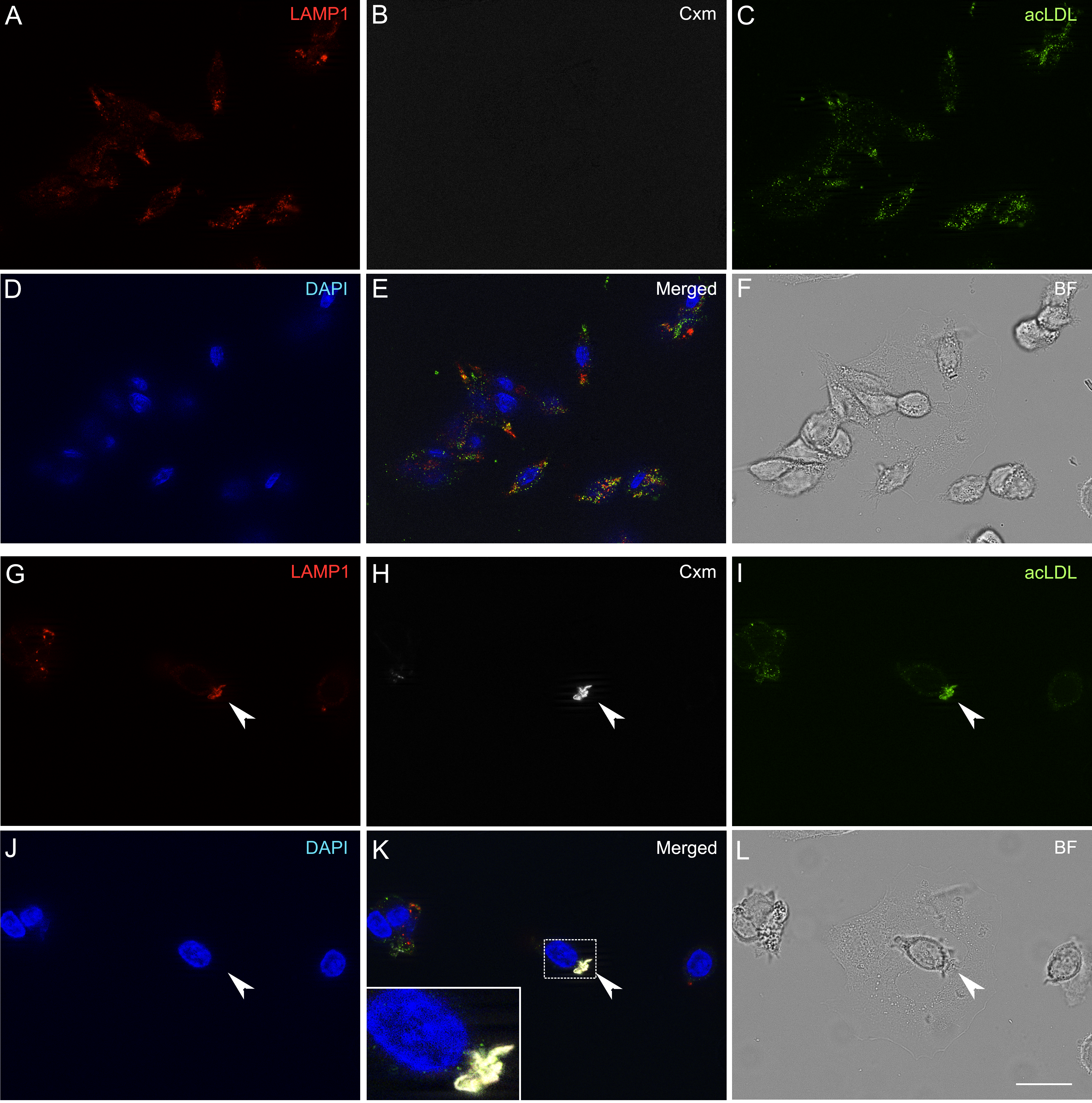

Supplement: Supplementary file 1 [file membranes-10-00091-s001.zip › Supplementary figures proof/Suppl Fig 1.tif]

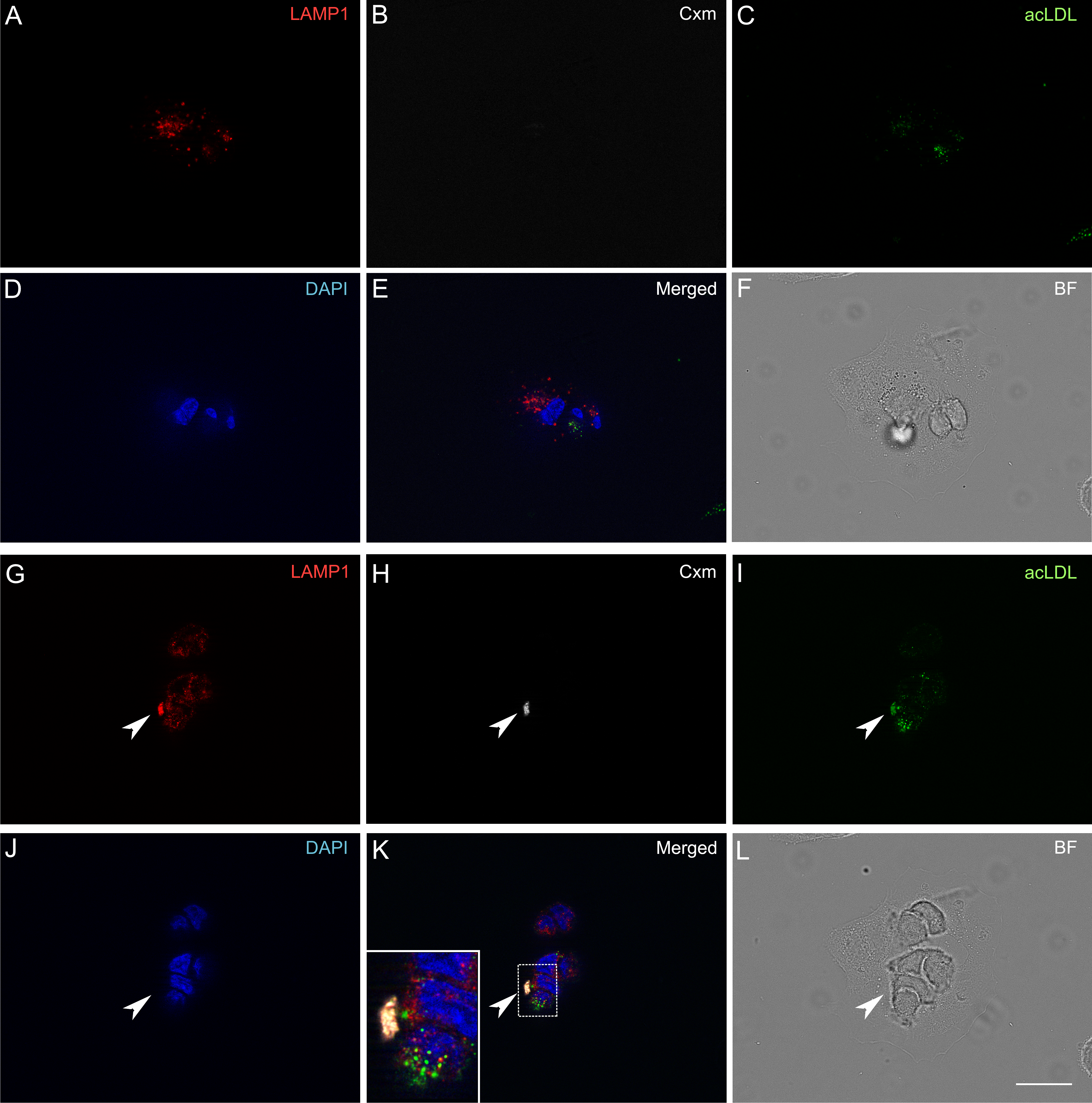

Supplement: Supplementary file 1 [file membranes-10-00091-s001.zip › Supplementary figures proof/Suppl Fig 2.tif]
